# Optimization and Evaluation of Magnetic Bead Separation Combined with Matrix-Assisted Laser Desorption/Ionization Time-of-Flight Mass Spectroscopy (MALDI-TOF MS) for Proteins Profiling of Peritoneal Dialysis Effluent

**DOI:** 10.3390/ijms15011162

**Published:** 2014-01-16

**Authors:** Na Guo, Qiong Wen, Zhi-Jian Li, Ri-Cong Xu, Fen-Fen Peng, Xue-Qing Yu

**Affiliations:** 1 Department of Nephrology, The First Affiliated Hospital, Sun Yat-sen University, 58^th^ Zhongshan Road II, Guangzhou 510080, China; E-Mails: guona@sysucc.org.cn (N.G.); wenqiong80@gmail.com (Q.W.); lizhijianlzj@sina.com (Z.-J.L.); xrc224@126.com (R.-C.X.); doctorpff@163.com (F.-F.P.); 2 Key Laboratory of Nephrology, Ministry of Health, Guangzhou 510080, China

**Keywords:** peritoneal dialysis, proteomics, matrix-assisted laser desorption/ionization time-of-flight mass spectrometry (MALDI-TOF MS)

## Abstract

Peritoneal dialysis effluent (PDE) potentially carries an archive of peptides relevant to pathological processes in abdominal and surrounding tissues. Magnetic beads and matrix-assisted laser desorption/ionization time-of-flight mass spectrometry is one such approach that offers a unique tool for profiling of peptides, but this approach has not been used in the PDE analysis. In this study, we developed a strategy for screening PDE proteins <15 kDa and applied this technique to identify potential biomarkers for peritonitis. We examined four kinds of magnetic beads, including a carbon series (C3, C8), weak cation exchange (WCX) and immobilized metal-affinity chromatography (IMAC-Cu) beads. Samples processed with IMAC-Cu magnetic beads consistently showed more MS signals across all beads within the measured mass range. Moreover, there was no difference in the number and morphology of MS signals between concentrated and unconcentrated samples. The PDE peptidome pattern, based on a panel of 15 peaks, accurately recognized peritonitis PD patients from peritonitis-free patients with sensitivity of 90.5% and specificity of 94.7% respectively. Therefore, IMAC-Cu magnetic beads and unconcentrated samples can be used as a fast and cost-effective approach for sample preparation prior to more in-depth discovery of predictive biomarkers of disease in patients on dialysis.

## 1. Introduction

Peritoneal dialysis (PD) is one of the main treatments for patients with end-stage renal disease (ESRD) [1,2] that allows solute and fluid exchange between peritoneal capillary blood and dialysis solution in the peritoneal cavity [3]. The dialysis membrane consists of the vascular wall, the interstitium, the mesothelium, and adjacent fluid [1–3]. Using the peritoneum as a dialysis membrane, dialysis solution continuously circulates through the abdominal cavity and peritoneal capillary. So, the peritoneal dialysis effluent (PDE) contains peptides and proteins leaked from blood, as well as those secreted by peritoneal cells and inflammatory cells, which play critical roles in many physiopathologic processes [4]. For PD-related peritonitis, the culture negative rate is typically >20% [5,6] and requires 24–48 h to obtain results to discriminate different types of peritonitis at the initial time which seems to be important for therapy. Analysis of the PDE proteome may detect changes that reflect issues with pathological processes in the abdominal cavity and peritoneal transport function. Furthermore, PDE is simply and non-invasively obtained. Thus, proteomic analysis of PDE may be a useful tool in the prediction, early diagnosis, treatment monitoring, and prognostic assessment of PD patients.

A variety of proteomic approaches have recently been used to characterize the peptide and protein composition in PDE. Most of these proteomic technologies are centered on the implementation of mass spectrometric (MS) techniques couple with several analytical techniques such as two-dimensional gel electrophoresis (2D-GE) or liquid chromatography (LC). While these approaches provide a large amount of data and identify hundreds of proteins, they are generally very time-consuming and hence restrictive in the number of comparative samples that can be analyzed. Magnetic bead enrichment in combination with matrix-assisted laser desorption/ionization time-of-flight mass spectroscopy (MALDI-TOF MS) technology (Bruker Daltonics, Bremen, Germany) is an approach that offers a unique platform for high-throughput protein profiling of complex biological samples such as body fluids [7–9].

Functionalized magnetic bead-based purification was developed to reduce costs and make proteomic procedures suitable for general analysis. This method uses different chemical chromatographic surfaces on an outer layer of magnetic beads to selectively purify certain subsets of proteins, allowing unbound impurities to be removed by washing with buffers [10,11]. Proteins bound to the magnetic beads are then eluted, diluted, and directly analyzed by MALDI-TOF MS. Genetic algorithms (GA) are then used to align and integrate hundreds of mass data points from large numbers of samples, and can be used to process many samples in parallel. This approach is sensitive and fast; these are features essential for clinical use [12,13]. Furthermore, the cost of using this technology is low, and further protein identification can be performed from the eluted material without complex purification. Thus, magnetic beads-based enrichment approaches have the potential to capture and enrich low abundance, low molecular weight species [14,15].

The current study used magnetic bead separation and MALDI-TOF MS for screening peptides and small proteins between the 1–15 kDa mass ranges. To date there was no previous study to describe the application of this technology in PD and the PDE sample. Proteomic analysis of PDE can be considered as a sophisticated process because PDE has a diluted protein concentration, high levels of salts and glucose, and more importantly, contains high-abundance proteins, including albumin and IgG, which interfere with the identification of proteins with low abundance profiles. Therefore, the aim of this study is to develop an optimal method to prepare reproducible and consistent separation of PDE proteome in the low molecular weight region.

## 2. Results

### 2.1. The Protein Profiles of PDE Samples Using Different Magnetic Beads

Profiles for PDE samples were compared across all four magnetic bead treatments (C3, C8, WCX and IMAC-Cu). The number and resolution of MS signals was used to determine the optimal magnetic bead to be adopted for a PDE profiling strategy. We first used 20 samples (10 from patients with peritonitis (PD + P) plus 10 from peritonitis-free patients (PD − P)) for screening. The fingerprint pattern and intensity varied for different bead types and were dependent on the sample. Approximately 100–200 MS signals were produced after fractionation by C3, C8, WCX or IMAC-Cu beads.

**Figure 1.**
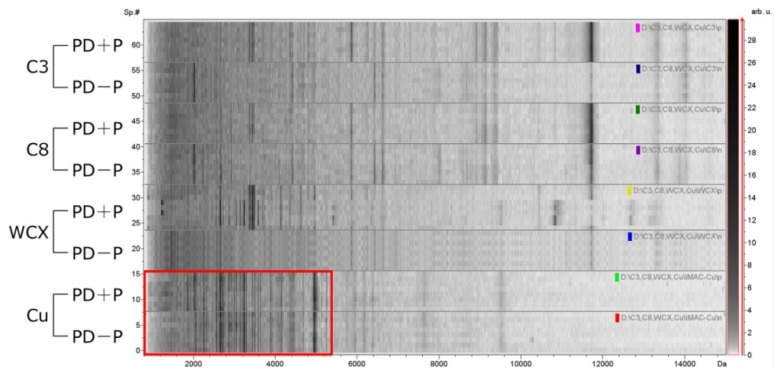
Protein profiles obtained with various chemical affinity beads. Peptides and proteins were captured by magnetic bead-based technology using C3, C8, weak cation exchange (WCX), and immobilized metal-affinity chromatography (IMAC-Cu) beads. In each panel, the compiled protein spectra from the PD + P and PD − P samples for each bead type are shown with the density plots of individual peritoneal dialysis effluent (PDE) profiles. *X*-axis, the calculated molecular mass (*m*/*z* values); *Y*-axis, relative intensity of specific samples. The differential density along the *x*-axis represents the specific peptide that distinctly presents in the samples. IMAC-Cu shows adequate protein profiles across all beads (labeled with a red frame).

Figure 1 shows representative spectra of proteins retained by the four magnetic beads. The carbon-series beads yielded profiles with the fewest signals, and the profiles show certain minor differences between C3 and C8 beads. WCX or IMAC-Cu beads both yielded a marked horizontal differentiation than the carbon-series; however, WCX beads did not produce as many unique signals as IMAC-Cu (Table 1). Thus, copper beads were chosen for further study of patient samples but optimization of PDE-matrix mixtures was required. After purification by IMAC-Cu magnetic beads, PDE was diluted with specific elution solutions. After analysis of 5-, 10-, and 20-fold dilutions with MS matrix solution, a 10-fold dilution yielded the best profiles.

### 2.2. Effects of Concentration on Proteins Separation

**Figure 2.**
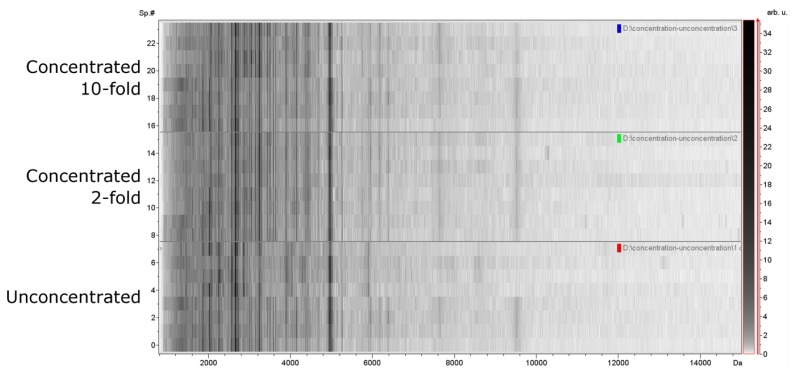
Protein profiles obtained with concentrated and unconcentrated samples. *X*-axis, the calculated molecular mass (*m*/*z* values); *Y*-axis, relative intensity of specific samples. Based on total signals count, unconcentrated samples consistently demonstrated a similar number of resolved fingerprints within the measured mass range of 1–15 kDa when compared with concentrated samples.

To determine whether the concentration of PDE interfered with proteins profiling, we compared the PDE protein profiles before and after concentration purified by IMAC-Cu beads. Purified PDE specimens were concentrated 2- or 10-folds before analysis. In general, the mass signals in the unconcentrated samples were similar to signals acquired from concentrated samples (Figure 2), and there was no difference statistically between the three groups (Table 2).

### 2.3. Reliability and Reproducibility of Protein Profiling Using IMAC-Cu Beads from Unconcentrated PDE Samples

For comparative studies, reliable and reproducible protein profiles must be obtained to ensure that the variation in spectra reflects biological differences in protein concentration rather than systematic variability. As such, accurate mass signal heights are necessary, and the technical variation of the profiles must be known. Therefore, we examined the precision of the assay using optimized conditions.

**Figure 3.**
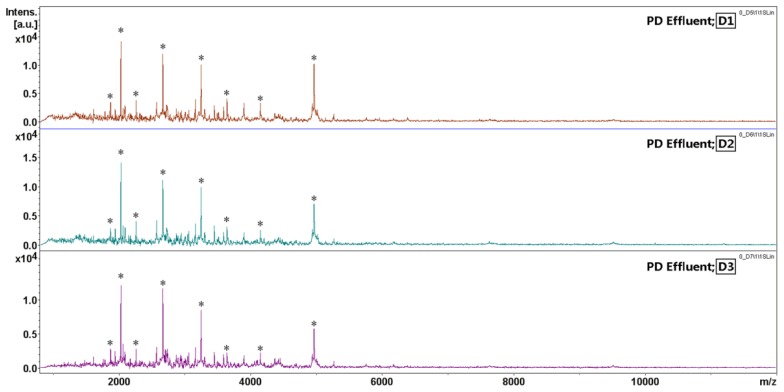
The intra-day reproducibility study for matrix-assisted laser desorption/ionization time-of-flight mass spectroscopy (MALDI-TOF MS) measurement. The same PDE sample was processed with IMAC-Cu in a single vial and spotted three times on SCOUT 600 μm AnchorChip target positions D1, D2, and D3. Eight MS signals with high, medium, and low abundances within the mass range of *m*/*z* 1–15 kDa were randomly selected for the variability assessment. The results are shown in Table 3. ^*^ selected MS signals.

The intra-day reproducibility of PDE MALDI-TOF MS spectral fingerprints was evaluated. The PDE sample was spotted three times on SCOUT 600 μm AnchorChip at target positions D1, D2, and D3. The PDE fingerprints with high, medium, and low abundance signals within the mass range of *m*/*z* 1–15 kDa were randomly selected and marked with asterisks for a variability assessment (Figure 3). Acquired spectra from replicate analyses (*n* = 3) exhibited sufficient reproducibility for semi-quantitative analysis. The variability of each MS signal area was measured by the coefficient of variance (CV). Despite varying peptide masses and spectrum intensities, the CVs were 4%–23% with an average intra-day variability of 10% (Table 3), which indicates that results produced by this method are highly reproducible for the analysis of PDE samples.

**Figure 4.**
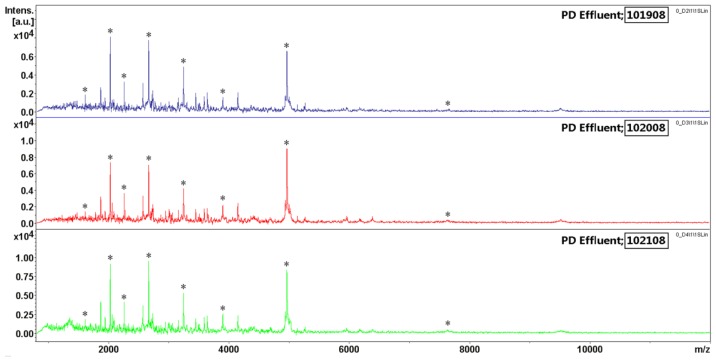
The inter-day reproducibility study for MALDI-TOF MS measurement. The sample was split into three vials and processed on different days (marked 101908; 102008; 102108). The variability in signal intensity was estimated across eight MS signals of high, medium, and low abundances within the mass range of *m*/*z* 1–15 kDa. The ratios of the signal intensities are listed in Table 4. ^*^ selected MS signals.

The inter-day reproducibility study, including sample processing and mass spectral analysis, was evaluated on three different days (Figure 4); variability was 8%–33% with an average CV of 22% (Table 4). Overall, both the inter- and intra-day CVs obtained indicate that the processing procedure was reliable and reproducible. These procedures minimize the systematic bias introduced by plates and time of processing. We also found that distinctive PDE fingerprints were concentrated in the mass range of 2–6 kDa, which meant that the MALDI-TOF MS is capable of screening lower-mass proteins or peptides.

### 2.4. Construction and Evaluation of the Classification Model for Recognizing Peritonitis

**Figure 5.**
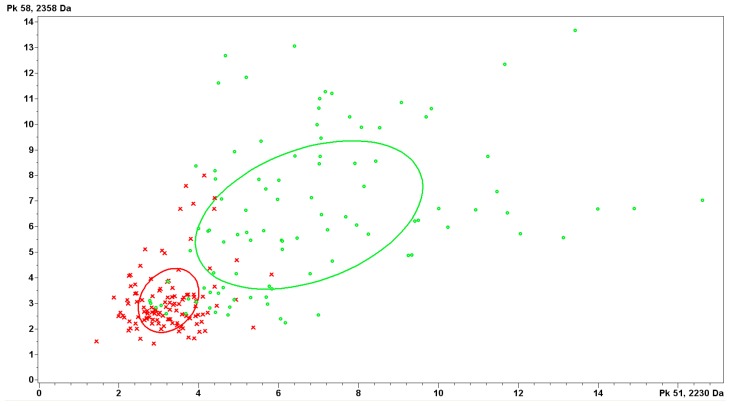
Discrimination features of peptides with *m*/*z* 2230 (*x*-axis) and 2358 (*y*-axis) between the patients with peritonitis (**red cross**) and control group (**green circle**).

By application of IMAC-Cu combined with MALDI-TOF MS, we analyzed the peptidome pattern between PD + P and PD − P. The spectra were analyzed by the Genetic Algorithm (GA) to generate a classification model which was based on 15 peaks different statistically between groups, with masses ranging from 1 to 12 kDa (Table 5). Within them, the top two discriminating peaks of 2230 and 2358 Da could distinguish peritonitis samples from control ones effectively (Figure 5). Further, the spectra from an independent testing set (*n* = 40, 20 from PD + P plus 20 from PD − P) were used for external validation of the GA-based model. The model evaluation and clinical diagnosis for each sample were compared (Table 6). The model could recognize peritonitis samples with a sensitivity of 90.5% (19/21) and specificity of 94.7% (18/19) respectively.

## 3. Discussion

Although some progress has been made in the proteomic analysis of PDE in the past several years, there was no previous study describing PDE proteome using the magnetic bead separation coupled with MALDI-TOF MS identification technology. In the present study, our results demonstrated that IMAC-Cu beads prepared with unconcentrated samples consistently showed more MS signals across all magnetic beads investigated with good reproducibility and credibility. According to this strategy, a classification model was constructed successfully, which could discriminate peritonitis patients from controls with high accuracy.

By using different types of beads, different subsets of proteins in a protein mixture can be captured. It has been successfully applied to the analysis of a wide range of samples such as serum, plasma, cell lysate, cerebrospinal fluid and urine. In this study, we selected beads according to the binding capacity, expressed as number of MS signals observed in MALDI-TOF spectrum, and showed that IMAC-Cu was the best performance extraction phase. Carbon-series magnetic bead whose external surface is modified with ligands of increasing carbon chain length (C1, C2, C3, C8 and C18) and with porous or non-porous surface uses hydrophobic interaction with proteins to enrich body fluid proteins with specific physicochemical properties, thus it is suitable only for a smaller range. Bruegel *et al.* used C8 beads to profile of human cerebrospinal fluid and obtained the highest number of reproducible peaks (more than 500) [16]. WCX magnetic bead was widely used in the purification of serum/plasma samples. Recently, a study was reported aiming at finding the best strategy to perform a comparative study of serum from breast patients and healthy control persons with magnetic beads with various functionalizations: C8, WCX and IMAC-Cu. The authors suggested using WCX bead as a starting approach since it resulted in the highest number of signals [17]. However, in our study it captured fewer PDE protein profiles than IMAC-Cu and IMAC-Cu beads demonstrated better capability to purify potential signal proteins in PDE, which was possibly because of the difference of characteristic between the serum and PDE samples. PDE contains large amounts of glucose; this characteristic may be different from the conventional body fluid in selecting magnetic bead type. However, the mechanism is not clear and further study still need to be investigated.

In previous studies, we recognized that most of the low abundance or small molecular weight proteins were negative when analyzed by traditional proteomics methods, such as 2D-GE, only allows the separation and quantification of proteins with molecular weight in a range of 20–300 kDa and lots of valuable diagnostic information may be lost [18–20]. Magnetic bead coupled with MALDI-TOF MS analysis provides optimal performance for *m*/*z* from 1 to 15 kDa, although it has no absolute upper analytical limit for *m*/*z* [21,22]. Larger peptides/proteins (>15 kDa) could also be found, while as *m*/*z* increases, resolution and mass accuracy progressively decrease. In the low m/z range, <1 kDa, there is higher background from ionized matrix molecules. Furthermore, many previous studies have already shown the great interest in the low molecular weight region as a source of diagnostic information, particularly substances smaller than 20 kDa [23]. So in this study, we focused on the analysis of peptides/proteins for *m*/*z* from 1 to 15 kDa. The larger peptides/proteins were also found but the data was not shown.

For sufficient sensitivity and specificity, current methods require the concentration of PDE samples, a process that can be time-consuming and labor-intensive, and has the potential to introduce errors. Furthermore, to achieve a highly concentrated sample, a large original sample volume is required, which may be not collected enough from clinical practice. Therefore, a method that can use unconcentrated PDE specimens while offering similar sensitivity and specificity would be desirable. As our results show, PDE specimens were concentrated to 2- and 10-fold before analysis, and the data obtained were similar to those for unconcentrated samples. The use of unconcentrated PDE specimens offers performance similar to concentrated sample but with considerably decreased errors, improved turnaround time (TAT), decreased cost of concentrators, decreased labor for technicians, and reduced test cancellations due to insufficient sample volume.

Systematic variability was observed across plates processed at different times to minimize systematic bias. The CVs obtained for both inter- and intra-day indicate that the processing procedure described here is reliable and reproducible. The reliability of the approach was emphasized by the low CVs suggesting that it can be adopted regardless of the systematic bias introduced by plates and time of processing.

Although the magnetic bead-based MALDI-TOF MS is a straightforward and robust platform for high-throughput protein profiling, there has been concern about its poor representation of the proteome that is not adequate for “deep” proteome analysis, particularly for proteins >15 and <1 kDa. However, this cannot be called the “Achilles’ heel” of magnetic beads-based MALDI-TOF MS. Each proteomic platform will reveal certain unique proteins not found with the other methodologies; therefore, for maximum proteomic coverage, a combination approach is recommended. Despite the current technical shortcomings of the magnetic bead-based MALDI-TOF MS technology, one could envisage its use as a strictly high-throughput screening tool to drive decisions on sample selection prior to more in-depth discovery of diagnostic markers.

## 4. Experimental Section

### 4.1. Sample Collection

PDE samples were collected from consenting PD patients (*n* = 60, 30 from patients with peritonitis (PD + P) plus 30 from peritonitis-free patients (PD − P)). The diagnosis of peritonitis was based on at least 2 of the following criteria: (1) clinical features of peritonitis (abdominal pain or cloudy dialysate); (2) leukocytosis in PDE (white blood cells count >100/μL, with at least 50% polymorphonuclear neutrophile granulocyte); and (3) positive Gram stain or culture from PDE [24]. Patients enrolled with peritonitis were diagnosed as *Escherichia coli* only, because *E. coli* related peritonitis is more common in Southern China. All participants had signed the written informed consent form before they enrolled this study.

All the samples were centrifuged within 30 min of collection at 10,000× *g* for 20 min to remove insoluble solids. Equal volumes (100 mL) of supernatants from the twenty samples were separated to two portions, one of which was concentrated by precipitation before treating with magnetic beads, while another was treated with beads directly. Supernatants were supplemented with the protease inhibitor PMSF (0.1 mg/mL) to inhibit proteolysis, frozen in aliquots and stored at −80 ºC.

PDE samples were subdivided into a training set and a testing set. The training set including 10 PD + P plus 10 PD − P samples was used for optimization of a strategy for screening PDE proteins and construction of classification model to recognize peritonitis patients. The testing set was used for external validation of the model.

### 4.2. Precipitation

PDE were precipitated using 75% acetonitrile (ACN) according to the previous reported methodology [25]. Cold ACN (−20 ºC) was added to the PDE samples to a final concentration of 75%. The mixture was stored overnight at −20 ºC and then centrifuged at 10,000× *g* at 4 ºC for 15 min to obtain a pellet. The supernatant was removed, and the pellet was air dried. All the pellets were dissolved with a solubilizing buffer containing 7 M urea, 2 M thiourea, 4% CHAPS, 2% (*v*/*v*) ampholytes and 40 mM DTT. The protein concentration was measured with the 2-D Quant kit (GE Healthcare, Piscataway, NJ, USA). By changing the volume of solubilizing buffer, we increased the protein concentration to 2- or 10-fold.

### 4.3. Pretreatment Using Magnetic Beads

PDE samples from each patient were test separately and repeated three times. They were purified with a reagent set with chemically-coated magnetic beads, including a carbon series (C3, C8), weak cation exchange (WCX) and immobilized metal-affinity chromatography (IMAC-Cu) beads (Bruker Daltonics, Leipzig, Germany). Samples were purified and isolated through three steps: binding, washing and elution. First, 5 μL beads, 40 μL binding solution (BS) and 80 μL PDE sample were added in a tube and mixed carefully and incubated for 5 min. The tube was then placed on a magnetic bead separation device for 5 min to collect the beads on the tube wall. The supernatant was removed and 100 μL magnetic bead washing solution (WS) was added and mixed thoroughly. After washing three times, the supernatant was removed and 10 μL magnetic bead eluting solution (ES) was added and the beads were collected on the tube wall in the separation device for 5 min. Finally, the clarified supernatant was transferred to a fresh tube and store at −20 ºC.

### 4.4. AnchorChip Spotting

The eluted sample was diluted 1:10 in matrix solution α-cyano-4-hydroxycinnamic acid (CHCA, 0.3 g/L in 2:1 ethanol:acetone), which was prepared daily. For analysis, 1 μL of the mixture was spotted onto an AnchorChip (Bruker Daltonics, Bremen, Germany) target and the droplet dried at room temperature before analysis.

### 4.5. Protein Profiling

Mass spectra were obtained using an Ultraflex III MALDI-TOF MS (Bruker Daltonics, Bremen, Germany) operated in positive-ion linear mode. All spectra were obtained randomly over the surface of the matrix spot. Before analysis, it was important to have the system quality controlled where 11 peptides (Bruker Daltonics, Leipzig, Germany) were used as an external standard preparation and the average molecular weight deviation was <100 μg/g; the standard preparation was used for calibration before data acquisition from samples. A coefficient of variability less than 30% indicated that the system ran well. Profile spectra were acquired from an average of 800 laser shots per sample. Peak *m*/*z* values or intensities were determined in the mass range of 1–20 kDa, focusing on mass range 1–15 kDa. Briefly, all spectra were processed by automatic baseline subtraction, peak detection, recalibration, and peak-area calculation according to the predefined settings.

### 4.6. Statistical Methods, Evaluation of Assay Precision

Each spectrum obtained from MALDI-TOF MS was analyzed using ClinProTools software (version 2.2; Bruker Daltonics, Bremen, Germany). Data analysis began with raw-data pretreatment, including baseline subtraction of spectra, normalization of a set of spectra, internal peak alignment using prominent peaks, and a peak-picking procedure. The criteria for MS signal detection were as follows: signal-to-noise (*S*/*N*) ratio > 3, a 2-Da peak width filter, and a maximum MS signal number of 300. The intensities of the MS signals of interest were normalized with the peak intensity of the internal standard. Whole data pretreatment was automatically performed using default settings, without any user interaction. The pretreated data were then used for visualization and statistical analysis in ClinProTools (Bruker Daltonics, Bremen, Germany). The MS signal area was used for quantitative standardization. A genetic algorithm (GA) (ClinProt 2.2; Bruker Daltonics, Bremen, Germany) was used for spectral analysis. This algorithm mimics natural evolution and is used to select the MS signals combinations that are most relevant for separation. Pattern determination is used to identify an optimal set of signals, which gives the best separating model determined upon the model generation spectra used, and validated on test spectra or by a cross-validation procedure. The parameter settings for model generation were as follows: the maximal number of best peaks was 15 and maximal number of generations was 50; automatic detection of the initial number of peak combinations was applied; the mutation and crossover rates were 0.2 and 0.5, respectively; use of varying random seed was not applied; and the number of neighbors was three. Diagnostic value of each MS signals was estimated by *t*-test. SPSS (version 13.0; IBM, Armonk, NY, USA) was used to analyze the coefficient of variation.

## 5. Conclusions

This study shown that the magnetic bead-based MALDI-TOF MS technology can provide a fast, robust, straight forward and reproducible profiling platform for measuring peptide fingerprints in the low molecular mass range of the PDE proteome. The use of IMAC-Cu beads with unconcentrated samples was sufficient to represent all assigned signals and, according to this strategy, an effective classification model could be constructed to recognize peritonitis patients. We anticipate its future usefulness in discovering potential biomarkers for PD-related diseases, as well as predicting the prognosis for individual patients.

## Figures and Tables

**Table 1.** Protein profiles obtained with various chemical affinity beads.

Groups	C3	C8	WCX	IMAC-Cu	*p* value
Peritonitis	71 ± 11	103 ± 19	175 ± 27	212 ± 25	0.012
Peritonitis-free	52 ± 16	87 ± 14	166 ± 23	205 ± 27	0.027

**Table 2.** Protein profiles obtained with various concentrations of PDE samples using IMAC-Cu beads.

Groups	Unconcentrated	2-fold	10-fold	*p* value
Peritonitis	207 ± 32	209 ± 27	205 ± 25	0.33
Peritonitis-free	201 ± 29	213 ± 21	211 ± 23	0.25

**Table 3.** Intra-day variability of MALDI-TOF MS after sample preparation with IMAC-Cu beads.

*m*/*z*	MS signal area			CV%

	D1	D2	D3	
1,865.9	6,164	5,424	5,872	6
2,022.3	27,771	30,127	31,744	7
2,251.8	13,354	16,763	15,057	11
2,660.9	71,469	64,529	66,094	5
3,154.7	21,513	23,140	18,290	11
3,241.8	67,561	66,391	67,204	9
4,146.9	21,106	19,212	19,683	5
4,963.7	41,587	28,283	44,451	23

Average CV%				10

CV, coefficient of variation.

**Table 4.** Inter-day variability of MALDI-TOF MS after sample preparation with IMAC-Cu beads.

*m*/*z*	MS signal area			CV%

	Day1 (101908)	Day2 (102008)	Day3 (102108)	
1607.4	1,629	1,767	1,089	24
2022.4	35,263	21,049	42,092	33
2251.5	12,093	13,520	15,774	13
2660.8	44,250	35,381	49,267	16
3241.3	29,019	22,306	34,462	21
3894.8	7,636	7,125	8,302	8
4963.2	51,412	62,928	87,357	27
7651.2	1,304	829	766	30

Average CV%				22

CV, coefficient of variation.

**Table 5.** PDE peptide peaks included in the genetic algorithm (GA)-based model to distinguish peritonitis patients from normal controls.

*m*/*z* a	MRI b ± SD in PD − P group	MRI b ± SD in PD + P group	*p* values c
1,436.74	3.69 ± 0.78	3.43 ± 0.72	0.0178
2,141.00	3.34 ± 0.70	3.69 ± 1.00	0.00935
2,230.33	3.60 ± 0.84	7.51 ± 3.16	<0.000001
2358.64	8.31 ± 3.13	9.45 ± 3.66	<0.000001
2,580.68	4.84 ± 2.23	7.51 ± 3.28	0.0276
2,661.40	31.45 ± 12.27	42.76 ± 11.16	<0.000001
3,472.38	2.66 ± 0.71	3.29 ± 0.80	<0.000001
3,864.19	2.73 ± 0.60	3.44 ± 0.67	<0.000001
4,001.97	2.21 ± 0.55	2.89 ± 0.75	<0.000001
4,036.69	2.91 ± 0.74	4.12 ± 1.01	<0.000001
4,266.15	2.20 ± 0.52	2.49 ± 0.53	0.000274
4,915.84	2.76 ± 0.75	3.30 ± 0.84	0.00000785
6,173.28	1.83 ± 0.56	1.46 ± 0.22	<0.000001
7,637.61	1.15 ± 0.27	1.31 ± 0.26	0.0000429
11,345.94	0.68 ± 0.13	0.70 ± 0.17	0.026

a *m*/*z*, indicated average mass;

b MRI, mean relative intensity;

c *p*-value was calculated by *t*-test (two tailed).

Values lower than 0.05 indicated statistical relevance.

**Table 6.** Evaluation of the PDE peptidome pattern for the testing.

Judgment of model	PD + P (*n* = 20)	PD − P (*n* = 20)	Total
PD + P	18	1	19
PD − P	2	19	21
